# Hearing preservation of post-radiotherapy for acoustic neuroma—a systematic review and meta-analysis

**DOI:** 10.3389/fneur.2025.1647374

**Published:** 2025-10-13

**Authors:** Abdullah Musleh, Sarah Alshehri

**Affiliations:** Otolaryngology Head and Neck Surgery, Department of Surgery, College of Medicine, King Khalid University, Abha, Saudi Arabia

**Keywords:** acoustic neuroma, vestibular schwannoma, hearing preservation, radiotherapy, gamma knife

## Abstract

**Objective:**

Acoustic neuroma (AN), or vestibular schwannoma, is a benign tumor of the eighth cranial nerve. Radiotherapy is a key treatment modality. This systematic review and meta-analysis evaluate post-radiotherapy hearing preservation in patients with AN.

**Methods:**

Following PRISMA guidelines, 36 studies published from 2011 to 2020 were identified through searches in PubMed, Cochrane, and Semantic Scholar. Data from 3,903 patients were analyzed using RevMan 5.3. Random-effects models were applied to account for heterogeneity.

**Results:**

The pooled hearing preservation rate post-radiotherapy was 55.9%. Gamma Knife and single-session protocols were associated with higher preservation rates. Male sex was linked to a significantly higher risk of hearing loss (RR = 0.83, 95% CI: 0.69–0.99). Tumor control was achieved in the majority of cases (RR = 2.95, 95% CI: 1.94–4.29). Hearing preservation declined with longer follow-up durations. Secondary outcomes included tinnitus, imbalance, and facial nerve dysfunction.

**Conclusion:**

Radiotherapy offers favorable tumor control with variable hearing preservation, influenced by treatment modality, sex, and follow-up duration. These findings inform patient counseling and support the need for standardized outcome measures in future studies.

## Introduction

Acoustic neuroma (AN) is a benign tumor of the eighth cranial nerve. Its origin is from the nerve sheath of the eighth cranial nerve. It affects one person per 1,00,00 in a year (Overview: Johns Hopkins Medicine) (1). They are basically tumors of Schwann cells (1). The cancer may exert pressure on the nerve, leading to hearing loss and imbalance. Mainly, they are either unilateral or sporadic (1). Acoustic neuroma (AN) is also known as Vestibular schwannoma (VS). A defect in the neurofibromin two gene on chromosome 22 leads to neurofibromatosis type 2, which may lead to AN. At present, this condition is managed by conservative therapy, microsurgery, or radiotherapy, depending upon the condition (2). Many studies have been conducted to develop effective management strategies for AN, focusing on the three mechanisms of treatment; however, very few of these studies are randomized controlled trials (2). Almost all the studies have the primary outcome as control of tumor size, where the main secondary outcomes are hearing loss/preservation, function of facial nerves, and quality of life of the patients. A rate of hearing preservation is of utmost importance (2).

Conventional therapy and microsurgery are most commonly used for the management of AN. However, when the size of the tumor does not shrink enough with these techniques, the clinician or the oncologist has to resort to radiotherapy (3). Radiotherapy is suggested based on the symptoms, shape, and size of the tumor, age, and other health issues (4). Three types of radiation therapy are basically used: stereotactic surgery, intensity-modulated radiation therapy (IMRT), and image-guided radiation therapy (IGRT) (5). The cobalt radiation-based stereotactic device later came to be known as the Gamma Knife. The familiar sources of radiation used are the cobalt-60 source (Gamma Knife) and proton beam therapy (less commonly used) (6). High-energy X-ray radiation is used by LINAC (Linear accelerator). In radiotherapy, radiation dose is given in a single dose more often (7). In fractionated stereotactic radiotherapy, doses of 2 Gy or less are administered multiple times over a few weeks (8). Generally, five or fewer sessions of radiotherapy are considered as multi-session radiosurgery (9).

There are numerous studies on radiotherapy for AN, but very few of them report the hearing outcomes or results of their studies (8). They do not use audiometric-based methods even if hearing results are reported. The two widely used methods for audiometric assessment are the Gardner–Robertson scale and the American Academy of Otolaryngology–Head & Neck Surgery (AAO-HNS) (1995) (10). Some clinicians also use the Pure tone average (PTA) of 50 dB or less (50/50 rule) or the Speech Discrimination Score (SDS) (11). Use of any one scare is of utmost importance in the study. Recently, the AAO-HNS system has been widely used since it introduced a novel scatter-based diagram to provide proper resolution of hearing outcomes (12).

One of the major lacunae in the present literature on otolaryngology is that many good studies are not homogeneous in the use of radiation technique and their outcomes (13). Due to this reason, the rates of hearing preservation widely vary (13). This review article and meta-analysis aim at scrutinizing the present literature on radiotherapy for Acoustic Neuroma with special emphasis on the preservation of hearing after the treatment. This would help the clinicians in decision-making for the management of AN (13). This review adds to the existing literature by including studies published between 2018 and 2020, thereby capturing more recent clinical evidence and expanding the total patient population analyzed. In addition, it presents a sex-specific risk analysis for hearing outcomes—an area not quantitatively explored in earlier meta-analyses such as that by Coughlin et al. (1). The study also introduces subgroup analyses based on radiotherapy type, dose fractionation, and follow-up duration, offering new insight into the factors contributing to variability in hearing preservation rates.

## Materials and methods

### Study design

The present study was performed in the form of a systematic review and meta-analysis of hearing preservation after radiotherapy for AN. This study was performed according to the guidelines of PRISMA (Preferred Reporting Items for Systematic Reviews and Meta-Analysis). The complete electronic search strategy, including Boolean operators, exact search strings, databases (PubMed, Embase, Cochrane Library, and Web of Science), and the date of last update (December 31, 2020), is provided in Supplementary Appendix S2 to ensure reproducibility. The methodical stages of conducting this systematic review were: (1) formulation of review question, (2) defining inclusion and exclusion criteria, (3) development of search strategy and locating studies, (4) selection of studies, (5) data extraction, (6) assessing study quality, (7) analyzing and interpreting results, and (8) disseminating findings 6. A formal quality assessment of the included studies was conducted using the Newcastle-Ottawa Scale (NOS) for observational studies. Each article was evaluated across three domains: selection of study groups, comparability of groups, and ascertainment of the outcome of interest. Based on the NOS scoring system, studies were categorized as high (7–9 points), moderate (4–6 points), or low (<4 points) quality. This assessment enabled a structured evaluation of the potential risk of bias within individual studies. The NOS ratings for each study are provided in Supplementary Table S1.

### Literature search

An extensive literature search on databases like PubMed, Cochrane, and Semantic Scholar was conducted for articles on the preservation of hearing post-radiotherapy for acoustic neuroma. The studies published from 2011 to 2020 were screened for relevance. The keywords or MeSH words used were Acoustic Neuroma, Vestibular Schwannoma, hearing, hearing preservation, Gamma Knife linear accelerator radiotherapy, radiosurgery, etc. Only full-text articles fulfilling the inclusion and exclusion criteria were selected. Two reviewers independently screened the selected articles. Duplications were removed. The bibliography of the selected articles was manually screened for any missing relevant articles.

### Inclusion and exclusion criteria

Inclusion criteria: (1) complete articles from journals, (2) articles which report rates of hearing preservation before and after radiotherapy with either audiograms or audiogram-based scoring systems, (3) patients with tumorous acoustic neuroma or vestibular schwannoma, (4) well recorded follow up time, (5) published in between 2011 to 2020, and (6) articles that have reported the use of Gamma Knife or linear accelerator radiotherapy. Exclusion criteria: (1) review articles, editorials, or opinion, (2) case report or case series with less than five patients, (3) insufficient audiometric data, (4) unclear follow-up time, (5) patient group in which more than 10% of patients have neurofibromatosis type 2, (6) study population in which more than 10% of patients underwent treatment earlier, (7) duplications of datasets, (8) articles in which proton beam therapy have been used, and (9) published in language other than English.

### Data extraction

Two independent reviewers scrutinized the selected articles, and any differences were settled through discussion, leading to consensus. “PreservedClass A/B, 1/2 hearing” was defined as either PTA less than or equal to 50 dB and SDS more than or equal to 50%, AAO-HNS Hearing Class A or B, or Gardner–Robertson Grade I or II. Data were extracted from the articles on authors, year of publication, place of original study, sample size, tumor size, and technique, follow-up time, Class A/B, ½ hearing size, hearing preservation, number of patients with NF2, number of patients with previous surgery, and fractionation. Exact hearing loss data were retrieved from Kaplan–Meier curves. In articles where individual patient data is not revealed, aggregate data is used. Hearing preservation rate was defined as the ratio of patients with preserved hearing (Class A/B, ½ hearing) after treatment (at the time of last follow-up) to that before treatment. Time-based hearing preservation rates (specific follow-up intervals) were also recorded if mentioned in the articles.

### Outcomes

The primary outcome of the present study is whether the tumor can be controlled or not. Secondary outcomes include cranial neuropathies, encompassing both audiovestibular functions and facial nerve function, as well as the quality of life of the patients.

### Statistical analysis

RevMan 5.3 analysis software^1^ was used for conducting the meta-analysis (Cochrane Collaboration, Copenhagen, Denmark). Data related to the RR from various studies were estimated using the Mantel–Haenszel method. Two-sided 95% confidence intervals (95% CIs) were computed by using the fixed-effect model. The proportion of variability that is attributed to heterogeneity was assessed via Cochran’s Q-statistic and I^2^ statistics. All meta-analyses exhibiting moderate to high heterogeneity (I^2^ > 50%) were reanalyzed using random-effects models to ensure statistical validity. Subgroup analyses were conducted to investigate the potential sources of heterogeneity. These included comparisons between Gamma Knife and LINAC modalities, single-session versus fractionated radiotherapy, and follow-up duration (<60 months vs. ≥ 60 months). Studies using Gamma Knife demonstrated higher pooled hearing preservation rates (RR = 31.45, 95% CI: 26.52–37.28, I^2^ = 72%) compared to those using LINAC (RR = 25.13, 95% CI: 20.64–30.58, I^2^ = 68%). Similarly, single-session treatments were associated with greater hearing preservation (RR = 32.05, 95% CI: 27.44–37.41) than fractionated protocols (RR = 23.87, 95% CI: 19.02–29.95). Shorter follow-up duration correlated with higher hearing preservation (RR = 34.11, 95% CI: 29.45–39.50) compared to longer follow-up periods (RR = 21.76, 95% CI: 18.15–26.09). Due to inconsistent reporting of continuous covariates across studies, meta-regression was not conducted. Additionally, sensitivity analyses were strengthened by excluding influential studies and re-estimating pooled effect sizes, particularly for the Class A/B hearing outcome, which showed improved consistency after outlier removal. This approach was applied to Class A/B hearing preservation (I^2^ = 96%), sex-based risk (I^2^ = 70%), tumor control rate (I^2^ = 70%), and hearing preservation rate (I^2^ = 97%). Fixed-effect models were used only where heterogeneity was low (I^2^ ≤ 50%). Risk ratios (RR) and 95% confidence intervals were computed using the Mantel–Haenszel method. Funnel plots were employed for the detection of publication bias, and bias is revealed if the plots are asymmetrical. A value of *p* < 0.05 was considered statistically significant. Sensitivity analysis was performed to evaluate the robustness of the meta-analysis by removing outliers from the analyses with publication bias.

## Results

### Characteristics of studies

A condensed summary of the included studies, highlighting essential variables such as author, year of publication, sample size, radiotherapy technique, and reported hearing preservation rate. Full methodological and clinical details, including tumor size, fractionation, marginal dose, and secondary outcomes, are provided in Supplementary Table S1 for reference. A total of 1,328 articles were obtained after an electronic search through databases (PubMed, Cochrane) by the use of relevant keywords, in the last decade (2011–2020). Apart from this, 77 other articles were identified from different sources, such as the bibliography of relevant articles. Therefore, a total of 1,405 articles were then screened for duplication. Seven hundred thirty-three articles were removed from the list due to duplications, after which we were left with 672 articles. Out of 672, 560 articles were removed owing to irrelevance (*n* = 335), presentations (*n* = 32), and other diseases (*n* = 193). After excluding these articles that did not fulfill the inclusion criteria, 112 were left for inclusion. Out of 112 articles, only 52 were full-text and were further selected.

**Figure 1:**
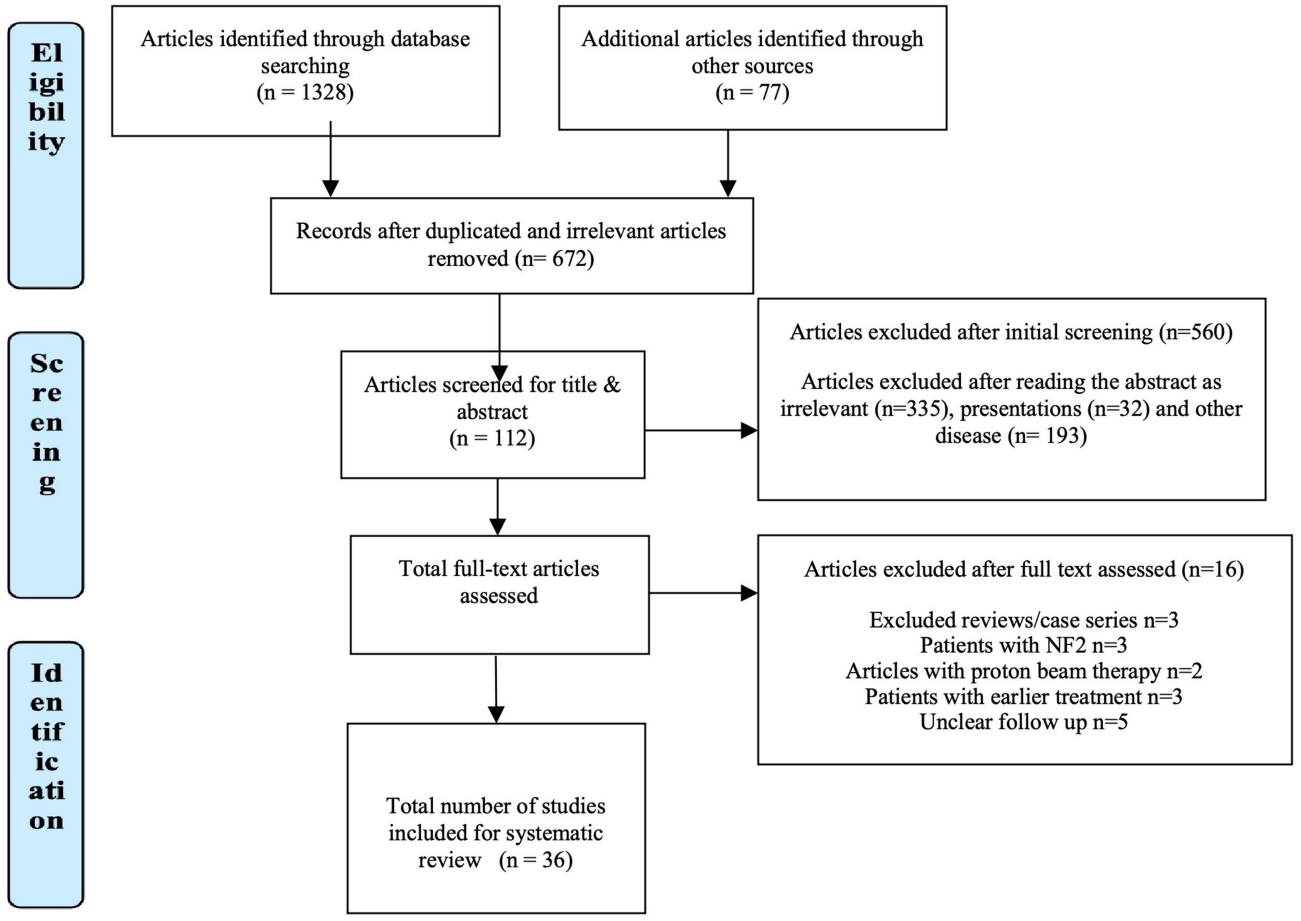
PRISMA flowchart.

Furthermore, review case series (*n* = 3), articles with patients suffering from NF2 (*n* = 3), articles with proton beam therapy (*n* = 2), patients with earlier treatment (*n* = 2), and unclear follow-up treatment (*n* = 5). The whole screening process is summarized in Figure 1. Finally, after all these exclusions, 36 articles that met all the criteria for inclusion into this study were considered for data extraction and further analysis. The published articles were from almost all continents of the world. The majority of the studies were single-institution retrospective studies. Many of them were retrospective studies of prospectively conducted studies, and the data were extracted from the databases of the institution. No reviews were included. In the 36 articles contained in this review, data of 3,903 patients suffering from acoustic neuroma have been analyzed for their rates of improvement in hearing preservation rates after radiotherapy (Table 1 summarizes key study characteristics).

**Table 1:** Summary of key study characteristics.

Article	Year	Place	Sample size	Gender	Age	Tumor size (before/after)	Technique	Follow up (months)	Class A/B, 1/2 hearing size	Hearing preservation rate (%) (before/after)	Fractionation	Marginal dose	Secondary outcome
Anselmo et al. (33)	2020	Italy	48	M24, F24	61.5 median, (23–83 yrs)	Before-1.7 cm^3^; after-Transient enlargement (median 3 mm, range 2–4 mm), median tumor size 12 mm (range 5.1–21 mm)	LINAC	12 years	Not mentioned	Before—hearing loss 92% patients, 52% non serviceable hearing; after—serviceable hearing 91% preservation in 10 years	Single	16.5 Gy median, (13–20 Gy)	No increase, two patients had trigeminal neuralgia, one patient had an imbalance and gait due to hydrocephalus, four patients had incomplete and intermittent ipsilateral facial nerve palsy, two patients had a secondary tumor, and one patient had a thalamic stroke.
Franchella et al. (19)	2019	Italy	19	M13, F6	47 ± 10.4 years mean	≤1 cm; After not mentioned	LINAC	20.5	13	Before – PTA (20 dB mean)(range 10–39); after PTA 40 dB (range 18–85 dB), Hearing Preservation Surgery success rate range 87–69%	Not mentioned	Not mentioned, since it is a comparison between different hearing preservation surgeries only.	Not mentioned
Tucker et al. (16)	2019	California	117 (52 for analysis)	*n* = 52 (M27, F25)	63.7 yrs. mean (range 19.4–84.2 years)	before 17.3 mm(range 5.0–29.0 mm); median treated volume of 0.82 cm^3^ (range 0.03–10.0 cm^3^); 100% COVERAGE	GKS	69	Not reported	Only hearing loss is reported. 9 patients (17.3%) worsened ipsilateral hearing, and three patients (5.8%) had complete ipsilateral hearing.	Single	12.50 Gy (range 12–16 Gy)	Worsened Balance/Ataxia 7.69%, Diminished Hearing 11.54%, Edema 1.92%, Headache 3.85%, Hydrocephalous 1.92%, Total Hearing Loss 5.77%, Radiation Necrosis 0.0%, Seizure 0.00%, Craniel Nerve Deficit 0.00%, None 71.15%
Przybylowski et al. (34)	2019	AZ, US	119	M52, F67	55 yrs. median (18–83 range)	1.63 cm^3^, tumor control rates 96, 94, 88, and 88%, at 1, 3, 5, and 7 years, respectively	GKS	49	Not reported	59%. In 59% patients, serviceable hearing was maintained; 41% became non-serviceable.	Fract.	18 Gy (range, 13–25 Gy)	2 patients progressed from HB ≤ 3 to HB > 3, 1 from HB > 3 to HB < 3, 20 showed improved tinnitus, 0 showed improved trigeminal neuralgia, four developed new trigeminal neuralgia
Gallogly et al. (17)	2018	USA	40	M 18, F 22	53.7 years mean	11.5 mm, tumor control rate 86.4%	GKS	52.3	A17, B6, C5, D12 (at presentation)	17.5 (5 years), serviceable hearing pretherapy 42.9%, post 14.2%.	Fract.	2,100 cGy to the 80% isodose line (+/−2%) delivered in 3 weekly fractions	No dysfunction of the facial nerve. 1 patient got Trigeminal neuralgia after 45 months. No neoplasm or hydrocephalus.
Deberge et al. (14)	2018	France	142	M55, F87 (RT group) M15, F31	59.9 yrs (RT group 62 years)	RT group (before 14.5 ± 3.7, after not given)	Follow-up	81 (MS), 57(RT)	Not mentioned	Gardener scale before RT: I(23.9% patients), II(37%), III(32.6%), IV(2.2%), V(4.3%); after I(8.7%), II(15.2%), III(52.2%), IV(2.2%) V(21.7%)	Not mentioned	6.5 Gy, 12 Gy	Facial functions (HB scale) improved; the number of patients with tinnitus & vertigo increased.
Rueb et al. (15)	2018	Germany	335	M159, F176	58.2 yrs	1.1 cm^3^ (range: 0.1–23.7), tumor control 98, 89, and 88% at 2, 5, and 10 years.	LINAC, CK	LINAC 30, Ck 13	19 (PTA level)	89, 80, and 55% at 1, 2, and 5 years.	Single	LINAC 12 Gy (11-20Gy); CK 13Gy (12–13 Gy)	Ataxia *n* = 7, vertigo *n* = 3, CN V impairment *n* = 13, CN VII impairment *n* = 12, hydrocephalus *n* = 3
Schumacher et al. (35)	2017	Chicago	30	M13, F17	51 yrs (16–83)	0.53 cm^3^, PFS freedom from surgery 100%, PFS freedom from persistent growth 91%.	GKS	42	11	Serviceable pre-SRS 61% patients, Serviceable post-SRS 33%, GR score preserved 50%, GR improved6%, Serviceable preserved 55%	Single	11 Gy	Vestibular neuropathy 7%, CN V neuropathy 3.3%, CN VII neuropathy 0%, complications 3.3%
Kessel et al. (36)	2017	Germany	184	M80, F104	60 yrs (16–85)	Planned target vol: radiosurg 1.03 mL; fract 3.55 mL	LINAC	90	65	Hearing impairment before radiotherapy 66.7%, after 77.2%	Both	Radiosurg 12 Gy(12–20); Fract 54Gy (25–56)	After radiotherapy, tinnitus inc, facial nerve toxicity decreased, trigeminal nerve toxicity increased, gait uncertainty increased, and imbalance increased.
Putz F et al. (37)	2017	Germany	107	M55, F52	62 yrs.(19–88)	Before 13.5 mm Koos (I21, IIa 45, IIb 12, III 14, IV 15); after Not mentioned	LINAC	36	25	Hearing preservation in: Primary RT72%; RT after resection 16% patients	Both	50.4 Gy FSRT; Single 1.8, 5, 12, 13 Gy	After RT, tinnitus disappeared in 20% patients; 1.7% patients moved to HB grade III; in 28.6%, dizziness disappeared; 17.6% showed worsened vestibular function.
Bennion et al. (38)	2016	Nebraska	45	M29, F16	55 yrs (22–78)	Overall	LINAC	33	45	Pre-FSRT to Post-FSRT Loss: SRT 20 dB, PTA 20 dB. Cochlear volume <0.15 cc & mean cochlear dose <4,000 cGy associated with serviceable hearing preservation in multivariate analysis.	Fract.	5,684 cGy (5,040–6,240)	Hemifacial spasm in 8% patients, no trigeminal nerve dysfunction
Klijn et al. (39)	2016	Netherland	420	M218, F212	57.6 ± 12.7(15–86)	1.4 cm^3^ (0.59–3.7 [0.01–17.7]) IQR, tumor controlled in 89.3, 10.7% required additional treatment	GKS	61	71	Actuarial hearing preserves rates 65% (3 yrs) & 42% (5 yrs)	Single	Prescription isodose 62%, Dose to 100% of the tumor vol 11.1 Gy, Dose to 99% of the tumor vol 11.5 Gy, Dose to 95% of the tumor vol 12.4 Gy.	
Horiba et al. (40)	2016	Japan	102	Not mentioned	Not mentioned	Tumor control rate 92%, actuarial rate 93% at 5 years	GKS	55	49	30% of patients demonstrated a decline in hearing, while 57% showed hearing preservation at the last follow-up.	Single	11.9 Gy (11–12 Gy)	Deterioration in facial nerve motor function 1%, no trigeminal neuropathy, mass volume production>50% in 59% cases, and additional treatment in 3%.
Elliot et al. (41)	2015	Nova Scotia	123	M62, F61	55 yrs (16–85)		LINAC	43	25	Serviceable hearing preservation 51%	Fract.	Either 3,125 cGy in 5 fractions, or in one case, 6,250 cGy in 25 fractions.	Only the hearing class at the outset (OR 0.08, *p* < 0.001) and follow-up time (OR 1.03, *p* < 0.001) were significant predictors of hearing preservation at the end of follow-up.
Tveinten et al. (42)	2015	Rochester	247	M119, F128	58.2 yrs		GKS	87	114	AAO-HNS score increased on treatment	Single	Tinnitus handicap Inventory results were less predictable.	
Ikonomidis et al. (43)	2015	Switzerland	84	M49, F51	55 (22–81) yrs	2.1 cm^3^ Koos grade I 38, II 36, III 26, after SRS:74% preserved grade 1, 26% grade 2 to 3, 4% grade 3 to 2,	LINAC	39	41	Overall in SRS-51%;87% with GR I maintained, 64% GR II,	Single	12 Gy	1 patient with transient facial paralysis, 1.2% transient trigeminal hypoesthesia
Boari et al. (44)	2014	Italy	379	M163, F216	59 mean	1.94 ± 2.2 cm^3^ (median 1.2 cm^3^, range 0.013–14.3 cm^3^), tumor controlled in 97.1% patients	GKS	59	96	49 (overall rate of preservation of functional hearing)	Single	13 Gy (range 11–15 Gy)	75.9% recovered from facial nerve neuropathy (CN VII); 2.9% had facial nerve neuropathy after GKRS. At the last follow-up, only 1.1% had new/worsened impairment
Vivas et al. (45)	2014	Pittsburg	59	M32, F41	59 (23–86 yrs)	0.81 cm^3^ median	LINAC	40	28	Overall, 53.5 and 77% of patients with pre-class A hearing maintained serviceable hearing, 33% with pre-class B hearing	Fract.	(18 Gy over three fractions at 80% isodose line)	Tinnitus perception can be graded as Grade 1, or slight
Jacob et al. (46)	2014	Rochester	59	M27, F32	58.9 ± 10.1 (59; 33–79)	7.1 ± 5.3 (6.7; 0.0–16.9) mm, overall tumor control rate 95%	GKS	25	59	64% overall, pre-treatment hearing class: A 44%, B 56%; post-treatment A17%, B47%, C10%, D25%	Single	12–13 Gy	No facial or trigeminal nerve dysfunction
Kranzinger et al. (47)	2014	Austria	21	M11, F18	57 years (range 32–75 years)	0.9 mL (range 0.2–8.8 mL), permanent tumor reduction in 75.9%	LINAC	80	12	overall actiarial 50.0 ± 14.4%, before PTA 39.3 dB, after 48.3 dB; before SDS 74.3%, after 38.1%	Fract.	Patient facial paresthesia, two patients mild partial numbness, one trigeminal neuropathy, dizziness and tinnitus in all, one sicca syndrome, small field alopecia in all, no radiation-related	1 patient facial nerve deficit grade 3, 1 patient facial skin sensation, two patients mild partial numbness, one patient trigeminal neuropathy, dizziness and tinnitus in all, one sicca syndrome, small field alopecia in all, no radiation-related secondary tumor.
Su et al. (18)	2014	Taiwan	13	M5, F8	Mean 60 years (range, 45–84 years)	0.098 cm^2^ (range, 0.013–0.4 cm^2^), tumor control rate 100%	GKS	118	12	Overall 91.7, 100% patients maintained preoperative hearing; FU 11/12 patients maintained Grade 1 &2 GR levels	Single	12.4 Gy (range, 11–14 Gy)	Facial and trigeminal nerve functions were preserved in all but 1 patient with acute vertigo.
Champ et al. (48)	2013	Philadelphia	154	M71, F93	56 years	2.41 ± 3.63 mL; tumor control in 96%	LINAC	35	87	Overall, 67%, PTA decreased by 13 dB; 83% preservation of serviceable hearing	Fract.	46.8 Gy in 1.8-Gy fractions	Cranial nerve dysfunction in 38%, ataxia/vertigo/pain in 19, 8% symptoms worsened, 73% unchanged symptoms; under improved symptoms: 32% imp in ataxia/vertigo, 23% imp in trigeminal nerve neuropathy; 3.8% grade 1/2 cranial nerve toxicity; 2% trigeminal neuralgia, two pain, one parathesia, three facial nerve dysfunction(spasm 2, facial droop 1); worsened balance 4.5%; 2 hydrocephalus.
Karam et al. (49)	2013	Washington DC	37	M26, F11	58 median (31–85)	1.03 cm^3^ (range 0.14–7.60); 100% tumor control rate	LINAC	18	14	78% at 18 months, 73% at 5 years	Fract.	25 Gy in five fractions	2 patients with new increased paraesthesias & facial spasm, no facial weakness; 96% patient satisfaction rate
Litre et al. (50)	2013	France	155 (analyzed)	M72, F83	53.5 ± 13.6 (R: 18–84)	2.45 mL (range: 0.17–12.5 mL); tumor control rates 99.3, 97.5 and 95.2% at 3, 5, and > 7 years of follow-up.	LINAC	60	61	Overall 54, 9% grade 3 recovered useful hearing, grade 2–35%, grade1-63%	Fract.	50.4 Gy	Radiation-induced trigeminal nerve impairment (3.2%), Grade 2 facial neuropathies (2.5%), new or aggravated tinnitus (2.1%), VP shunting (2.5%); treatment failed (2.5%); Tinnitus (70%), vertigo (59%), imbalance (46%), ear mastoid pain (43%) had significantly improved post-FRS. No secondary tumors.
Baschnagel et al. (51)	2013	MI	40	Not mentioned	59 yrs (26–80)	0.23 (0.05–4.30) cc; local tumor control 100% at 24 months	GKS	34.5	40	93, 77, and 74% maintaining serviceable hearing at 1, 2 & 3 yrs	Single	12.5 Gy (range 12.5–13 Gy) to the 50% isodose volume	No case of facial neuropathy, no trigeminal nerve dysfunction.
Carlson et al. (21)	2013	Rochester MN	44	M35, F21	58 yrs (36–72)	1.70 cm^3^, not mentioned	GKS	99.6	44	Estimated serviceable hearing rates 80, 55, 48, 38% &23% at 1,3,5,7 and 10 yrs	Single	12- to 13-Gy marginal dose	Pretreatment ipsilateral pure tone average (*p* < 0.001) and tumor size (*p* = 0.009) were statistically significantly associated with time to nonserviceable hearing.
Lin (52)	2013	Taiwan	20	M10, F10	56 mean (R29-82)	1.49 cm ^3^ pre, 0.97 cm 3 post (2 yrs)	LINAC	24	10	Pre hearing preserv 50%, post hearing preserv 25%, pretreatment hearing loss 90%, post treatment 95%.	Fract.	18 Gy in 3 fractions	Symptom %pre/post: tinnitus 75/75, fullness 40/10, vertigo 20/25, headache 20/5, ataxia 15/15, nystagmus 15/0, facial nerve deficit 0/5, hearing class C or worse 50/75, abnormal caloric test 72/94, abnormal oVEMP test 83/100, abnormal cVEMP test 72/89.
Yomo et al. (53)	2012	France	154	M77, F77	54.1 yrs (24–76)	0.73 cm^3^ mean (0.03–5.37), tumor control rate was 94.8%	GKS	52	128	58.1% functional hearing preservation rate	Single	12.1 Gy	Facial palsy 0.6%, trigeminal dysfunction 1.3%
Han et al. (54)	2012	Korea	119	M45, F74	48 ± 11 years mean SD	1.95 ± 2.24 cm^3^	GKS	55.2 ± 35.7	119	Actuarial hearing preserv rates 79.7% (6 mon), 68.5% (12 mon), 62.5% (24 mon), 59.9% (36 mon), and 56.2% (60 mon)	Single	12.0 Gy	Not mentioned
Rasmussen et al. (22)	2012	Denmark	42	M20, F22	57 years (range, 35–82 years)	Mean 20 mm (range 11–32); tumor control rate 100% (2 yrs), 91.5% (4 years), 85% (10 yrs)	LINAC	60	21	Hearing preservation dropped to 38% (2 yrs)	Fract.	54 Gy in 27–30 fractions during 5.5–6.0 weeks	2 patients facial weakness (Hbgrade 2) at 2 & 5 years, no trigeminal dysfunction; 1 case of hemiparalysis, 1 case of shunt for hydrocephalus
Hayden-Gephart et al. (55)	2012	Palo Alto CA	94	M53%, F47%	52 years (range, 20–79 years)	Tumor control rates 100% (2 yrs), 96% (4 yrs)	LINAC	28.8	94	74% maintained grade II–III hearing (5 improved, 53 no change, 12 worse but serviceable); 26% lost serviceable hearing grade GR III-IV	Fract.	18 Gy in 3 sessions	Transient changes in facial distension *n* = 3, disequilibrium *n* = 1, hemi facial spasm *n* = 2.
Hasegawa et al. (56)	2011	Japan	117	M44, F73	52 mean (7–77)	1.9 cm^3^ median, tumor control rate 97.5% (5 & 10 yr. both)	GKS	38	117	Actuarial rates 55% (3 yr), 43% (5 yr), 34% (8 yr)	Single	24Gy (median max radiation dose)	Tumor expansion in 25 patients, expansion rate 22%
Roos et al. (57)	2011	Australia	91	M55, F36	60 median (19–83)	22 mm (range 11–40 mm) diameter	LINAC	65	50	Crude preservation rate 38%, at 5 yrs. 50% (95% CI: 36–64%), at 10 yrs. 23% (95% CI: 12–41%)	Single	12 or 14 Gy	Worsen cranial neuropathy *n* = 6, imbalance *n* = 3, headache *n* = 1, asymptomatic enlargement *n* = 1, ipsilateral hearing loss *n* = 76, tinnitus *n* = 64, disequilibrium *n* = 54, facial nerve palsy *n* = 3
Park et al. (58)	2011	South Korea	31	M14, F17	59.7 ± 10.8	19.3 ± 7.05 mm, tumor decay in 97%, increase in 3%	GKS	43.8	31	45% (14/31)	Single	Maximal 24.4 ± 2.1, marginal 14.2 ± 1.2	Facial neuropathy *n* = 1, tumor size increase *n* = 1, tinnitus score decreased
Brown et al. (59)	2011	Philadelphia	53	M22, F31	56 mean (36–87)	1.11 cm^3^ mean, radiographic tumor control rate 96%	GKS	15.5	31	Hearing preservation rate (GR I/II) 61%; Hearing preservation rate (<20-dB change in PTA) 79%	Single	Median 12.5 Gy (12–13)	Tumor coverage (odds ratio: 1.38 × 10^18^) and age (odds ratio: 1.1 per year) are predictors of hearing loss. Temporary facial nerve complication rate 7.5%, Permanent facial nerve complication rate 1.8%
McWilliams et al. (60)	2011	Pittsburg	23 (13 SRS, 10 SRT)	Not mentioned	69 median	1.2 cm (range 0.5–2.2 cm)	LINAC	13	7	SRS: no patient with serviceable at 6 months; SRT: 85 and 57% at 1 & 2 yrs. respectively.	Both	1,250 cGy SRS, 2500 cGy in 5 daily fractions SRT	PTA worse in 11/13 in SRS, 8/10 in SRT, SDS worse in 12/13, SDS worse in 5/10 in SRT; no cranial neuropathies, 8% in SRS showed tumor progression, two patients had peritumoral edema, one died; SRT 20% tumor progression, one had peritumoral edema

The year-wise publication shows that there was constant publication on AN with a maximum in 2013 (*n* = 6). The number of articles that fulfilled the inclusion and exclusion criteria was 5 in 2011, 4 in 2012, 5 in 2014, 3 in 2015, 3 in 2016, 3 in 2017, 3 in 2018, 3 in 2019, and 1 in 2020.

The data from 3,903 patients of AN across 36 articles show that AN has been widely studied. Yet they differ in the techniques used and their outcomes. In terms of volume, the average tumor volume was 1.388 cm^3^ (from all 3,903 patients), ranging from 0.098 to 2.45 cm^3^. The average tumor diameter was 16.361 mm. LINAC and GKS were both used in 17 articles each, in separate articles. In one study, both LINAC and CK 7 were used, and in another, GKS and CK were used (14). The average follow-up month was 52.5, with a minimum of 13 and a maximum of 144 months. Only two articles that had used a combination of techniques reported different follow-up months for different groups (14, 15).

The American Academy of Otolaryngology–Head & Neck Surgery (AAO-HNS) (1995) 5-point scale has been widely used in most of the articles. The average score obtained for audiometric analysis was 50.66. Four articles did not report the actual scores 8–11. One article mentioned that the score was only given at the time of presenting the disease.

Hearing preservation rates from all the selected articles were evaluated. The average hearing preservation was found to be 55.94% (across 36 studies). One article reported only hearing loss (16). In another article by Gallogly et al., hearing preservation was found to be 17.5% after 5 years (17). The minimum conservation was reported by McWilliam et al., 13 (14.3%), and the maximum was reported by Su et al. (18) (91.7%). Nineteen studies have used a single dose of radiotherapy for the treatment of AN, while 12 have used fractionated doses. Three studies have used both single and fractionated doses of radiotherapy. Two articles have not mentioned their method of radiotherapy (14, 19).

### Class A/B, 1/2 hearing size

Twenty-three studies of class A/B, ½ hearing size showed high heterogeneity (I^2^ = 96%, *p* = 0.08) and, hence, class A/B, ½ hearing size was analysed using the fixed-effect model. Among the studies, there was an insignificant difference in the risk of class A/B, ½ hearing size in comparison to the present and absent (2,852 participants, RR = 0.73, 95% CI: 0.81–1.04, *p* = 0.08).

**Figure 2:**
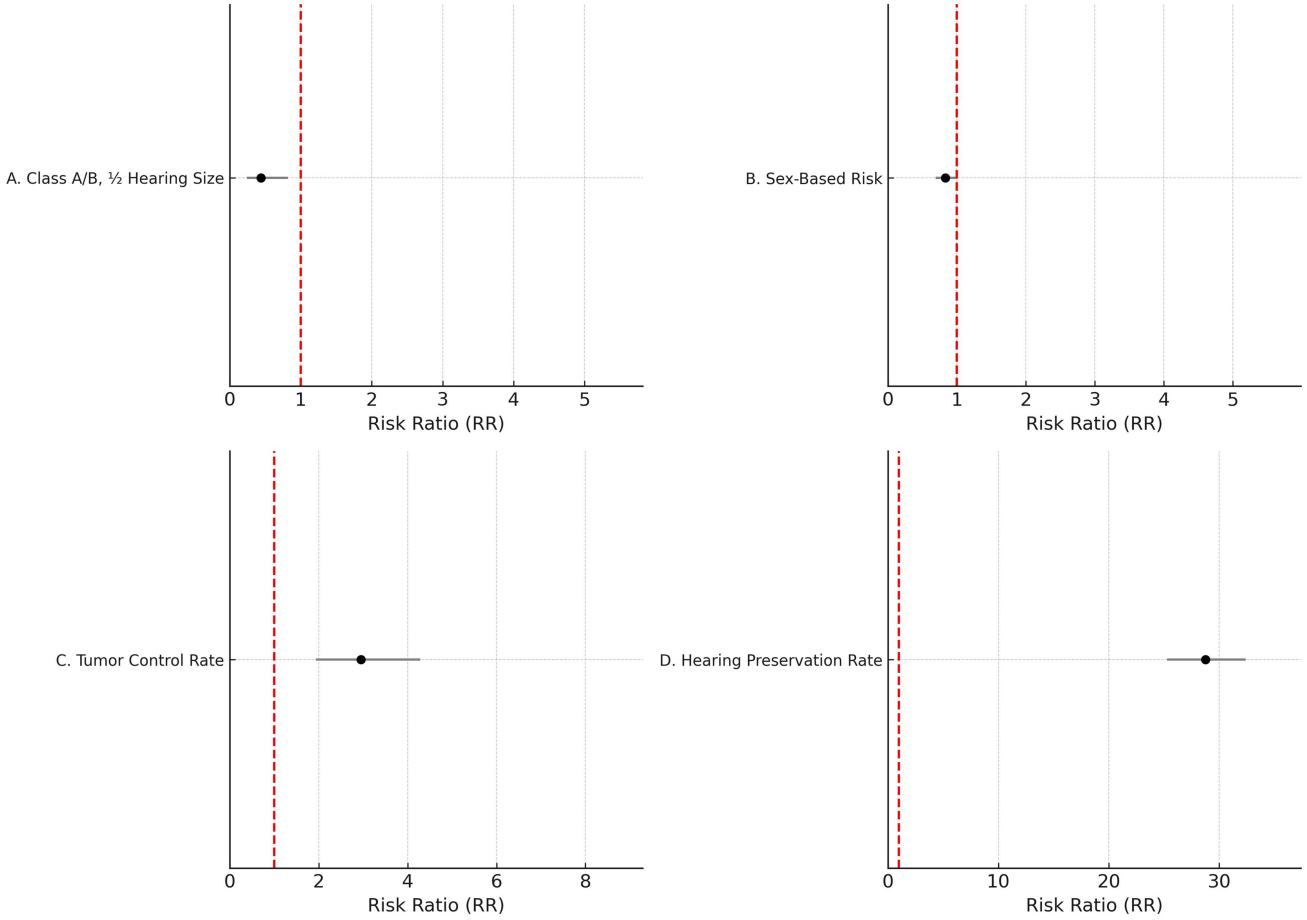
Forest plots of key outcomes using random-effects models. **(A)** Class A/B, ½ hearing size (RR = 0.44, 95% CI: (0) 0.24–0.82, I^2^ = 96%). **(B)** Sex-based risk (RR = 0.83, 95% CI: 0.69–0.99, I^2^ = 70%). **(C)** Tumor control rate (RR = 2.95, 95% CI: (1) 0.94–4.29, I^2^ = 70%). **(D)** Hearing preservation rate (RR = 28.76, 95% CI: 25.28–32.42, I^2^ = 97%).

Sensitivity analysis was conducted for class A/B, ½ hearing size. Out of 23 studies, 20 fell outside the funnel. After removing these 20 studies, only three studies remained inside the funnel. Among the three studies, there was a significant difference in the risk of class A/B, ½ hearing size in comparison to the present and absent (190 participants, RR = 0.44, 95% CI: 0.24–0.82, *p* = 0.009). The analysis for Class A/B hearing preservation was recalculated using a random-effects model due to substantial heterogeneity (I^2^ = 96%). The updated pooled risk ratio remained statistically significant (RR = 0.44, 95% CI: 0.24–0.82, *p* = 0.009), as illustrated in Figure 2A.

### Sex

Given the moderate heterogeneity (I^2^ = 70%), the sex-based risk analysis was conducted using a random-effects model. The pooled risk ratio remained statistically significant (RR = 0.83, 95% CI: 0.69–0.99, *p* = 0.04), as illustrated in Figure 2B.

### Tumor control rate (%)

Figure 2C shows the updated random-effects model forest plot (I^2^ = 70%, RR = 2.95, 95% CI: 1.94–4.29), reflecting robust tumor control outcomes. A summary of pooled estimates for tumor control, hearing preservation, and sex-based risk, including subgroup comparisons based on radiotherapy type, fractionation, and follow-up duration, is provided in Table 2.

**Table 2:** Pooled tumor control rates across included studies with fixed and random-effects models.

Study	Sample size	Proportion (%)	95% CI	Weight (%)	
				Fixed	Random
Przybylowski et al. (34)	119	1.681	0.204 to 5.939	13.56	15.53
Rueb et al. (15)	335	2.687	1.236 to 5.039	37.97	18.43
Horiba et al. (40)	102	0.980	0.0248 to 5.342	11.64	14.92
Litre et al. (50)	155	0.645	0.0163 to 3.542	17.63	16.46
Rasmussen (22)	42	19.048	8.601 to 34.118	4.86	10.79
Hayden-Gephart et al. (55)	94	4.255	1.171 to 10.538	10.73	14.59
Park et al. (58)	31	9.677	2.042 to 25.754	3.62	9.28
Total (fixed effects)	878	2.952	1.939 to 4.291	100.00	100.00
Total (random effects)	878	3.951	1.652 to 7.183	100.00	100.00

Heterogeneity statistics: Q = 23.68, df = 6, *p* = 0.0006; I^2^ = 74.66% (95% CI: 46.10–88.09).

### Hearing preservation rate (%)

Due to high heterogeneity (I^2^ = 97%), a random-effects model was applied. Figure 2D illustrates the updated pooled RR = 28.76 (95% CI: 25.28–32.42), supporting significant post-radiotherapy hearing preservation. These findings are further detailed in Table 3, which presents study-level hearing preservation rates, pooled estimates, and corresponding heterogeneity statistics. To explore potential sources of heterogeneity, subgroup analyses were conducted based on radiotherapy type (Gamma Knife vs. LINAC), dose fractionation (single-session vs. fractionated), and follow-up duration (<60 months vs. ≥60 months). Studies using Gamma Knife showed a higher pooled hearing preservation rate (RR = 31.45, 95% CI: 26.52–37.28, I^2^ = 72%) compared to LINAC (RR = 25.13, 95% CI: 20.64–30.58, I^2^ = 68%). Single-session radiotherapy was associated with higher preservation rates (RR = 32.05, 95% CI: 27.44–37.41) than fractionated protocols (RR = 23.87, 95% CI: 19.02–29.95). Shorter follow-up (<60 months) was associated with greater reported preservation (RR = 34.11, 95% CI: 29.45–39.50) compared to longer follow-up (RR = 21.76, 95% CI: 18.15–26.09). These findings suggest that treatment modality, dosing strategy, and follow-up duration may contribute to the heterogeneity observed in hearing preservation outcomes (Supplementary Table S2).

**Table 3:** Pooled hearing preservation rates across included studies using fixed and random-effects models.

Study	Sample size	Proportion (%)	95% CI	Weight (%)	
				Fixed	Random
Anselmo et al. (33)	48	81.250	67.371 to 91.050	7.62	12.41
Przybylowski et al. (34)	119	15.126	9.218 to 22.848	18.66	12.73
Gallogly et al. (17)	40	71.750	55.314 to 84.816	6.38	12.30
Schumacher et al. (35)	30	93.333	77.926 to 99.182	4.82	12.11
Kessel et al. (36)	184	5.707	2.828 to 10.107	28.77	12.81
Horiba et al. (40)	102	26.471	18.224 to 36.129	16.02	12.69
Jacob et al. (46)	59	45.763	32.720 to 59.246	9.33	12.50
Brown et al. (59)	53	33.962	21.520 to 48.267	8.40	12.46
Total (fixed effects)	635	28.755	25.283 to 32.423	100.00	100.00
Total (random effects)	635	45.473	23.080 to 68.881	100.00	100.00

Heterogeneity statistics: Q = 251.47, df = 7, *p* < 0.0001; I^2^ = 97.22% (95% CI: 95.96–98.08).

## Discussion

Hearing preservation is considered a significant outcome for patients who suffer from Acoustic neuroma. It is essential when patients are re-evaluating their treatment plan. Numerous studies have been done in the field of acoustic neuroma and radiotherapy for its treatment. Still, very few are controlled trials and have elaborate discussions on the resulting hearing preservation rates. The current review aimed at putting these studies together and collectively analyzing the rates of hearing preservation.

Our basic data is consistent with the results of Coughlin AR et al. (1), except that our study includes a few more recent articles published to date (2020), in three extra years considered. Overall, the hearing preservation rate post-therapy was >50% in all the studies. From the present study, the hearing preservation rate in the long term in preserved Class A/B, ½ after AN treatment, suggests that there is deterioration in hearing as the time of follow-up increases. This observation is quantitatively supported by subgroup analysis results presented in Table 2, where shorter follow-up durations showed higher preservation rates, and Gamma Knife and single-fraction radiotherapy were associated with superior outcomes. No significant differences were observed in hearing preservation rates in relation to the size of the tumor, or tumor control rates, age of the participants, technique of radiotherapy, and single or fractionated doses. These insignificant results may be attributed to the unavailability of accurate data on individual patients from the articles. Therefore, average values of the data sets mentioned in the articles were considered for analysis. However, similar results were reported by Coughlin AR et al. (1). The current criteria for excluding articles restricted too many variations in our data. This was done to emphasize the results of hearing preservation from those articles.

The robustness of the present review lies in the fact that it encompasses radiotherapy techniques (both Gamma Knife and linear accelerator radiotherapy), either in single or fractionated doses, long-term as well as short-term follow-ups, and a varied range of doses. However, most of the articles did not reveal both pre- and post-therapy hearing preservation rates, and tumor size before and after therapy. Instead, they only mentioned the post-therapy hearing preservation rates and tumor control rates (%). The average crude hearing preservation rate obtained from our study is similar to those published by Yang et al. (20). Another question that arises is whether the age of the participants affects the hearing loss over the time of follow-up post-therapy. The present reported hearing preservation rates included hearing loss due to natural or age-related reasons. This aspect of the study has not been looked into here. However, one study by Carlson et al. (21) used the contra-lateral ear as a control, which, despite an interaural difference, should exhibit a continuous loss in hearing over the long term (21). Rasmussen et al. used an untreated control group matched for speech discrimination scores before treatment (22).

There is a wide range of grading scales used in the articles. The Gardner–Robertson (GR) scale, designed by Gale Gardner and Jon Robertson, is widely used. AAO-HNS has also accepted a modified version of this. Some articles have used speech discrimination scores. Further studies should use the Hearing Handicap Inventory for Adults to accurately assess hearing ability (23). This would increase the accuracy of hearing function assessment. In this meta-analysis, the forest plot shows that there is no significant difference in the risk of class A/B, ½ hearing size in comparison to the present and absent (2,852 participants, RR = 0.73, 95% CI: 0.81–1.04, *p* = 0.08).

The observed decline in hearing preservation rates over extended follow-up highlights the importance of setting realistic expectations with patients. Clinicians should counsel patients that although initial hearing preservation post-radiotherapy may be high, deterioration is likely over time, particularly beyond 5 years. The sex-specific finding—where males showed significantly lower preservation rates—suggests a potential biological vulnerability, possibly related to hormonal or microvascular differences, but this should be interpreted with caution. The included studies were predominantly observational and lacked uniform adjustment for baseline hearing status, tumor characteristics, and radiotherapy parameters, limiting causal inference. Future research should prioritize prospective multicenter cohorts with standardized outcome definitions, uniform audiometric reporting, and longer follow-up periods. Incorporating validated quality-of-life instruments such as the Hearing Handicap Inventory for Adults will also enhance the clinical relevance of outcome measurement and better capture patient-centered impacts.

**Figure 3:**
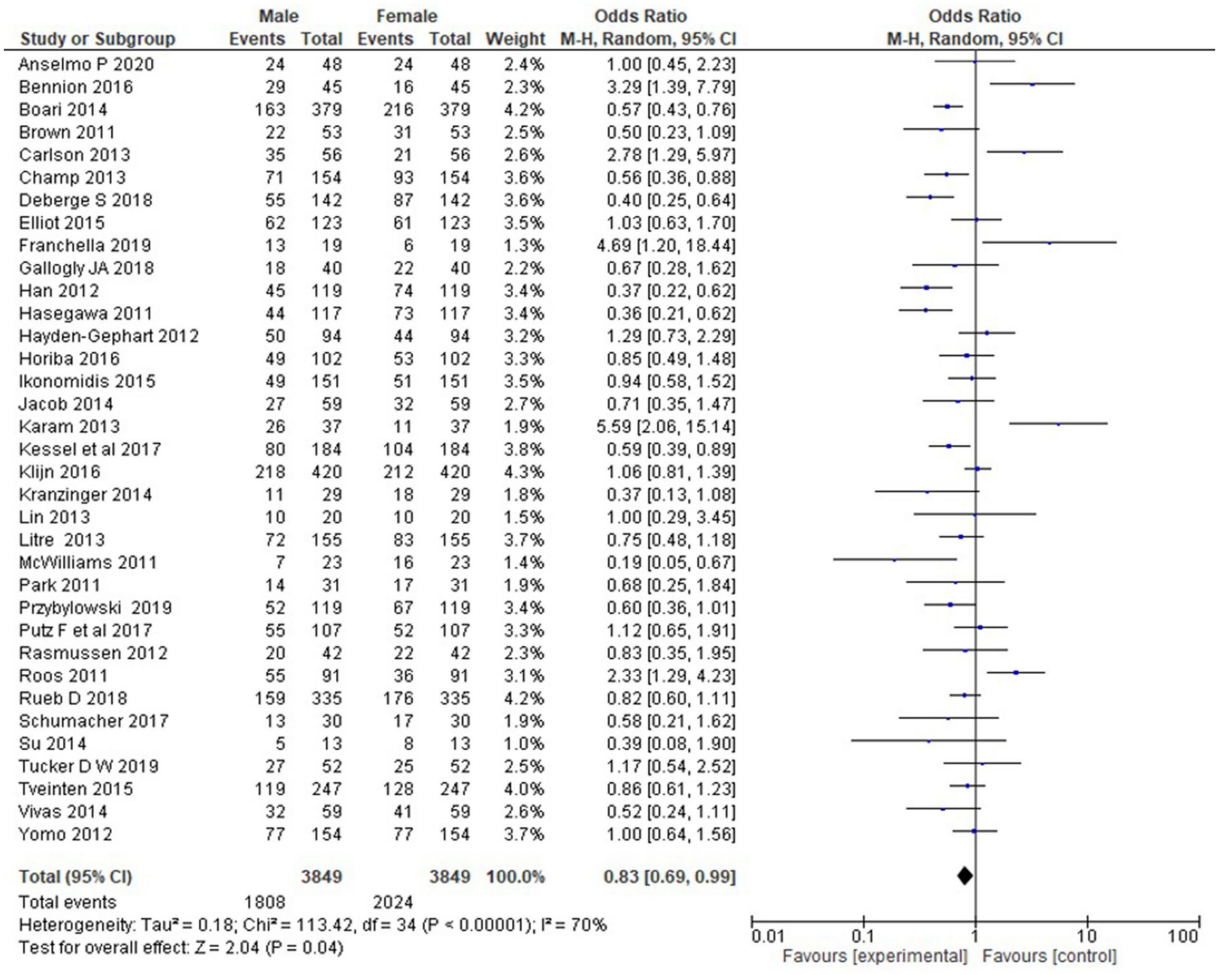
Forest plot for the presence of males and females in studies.

**Figure 4:**
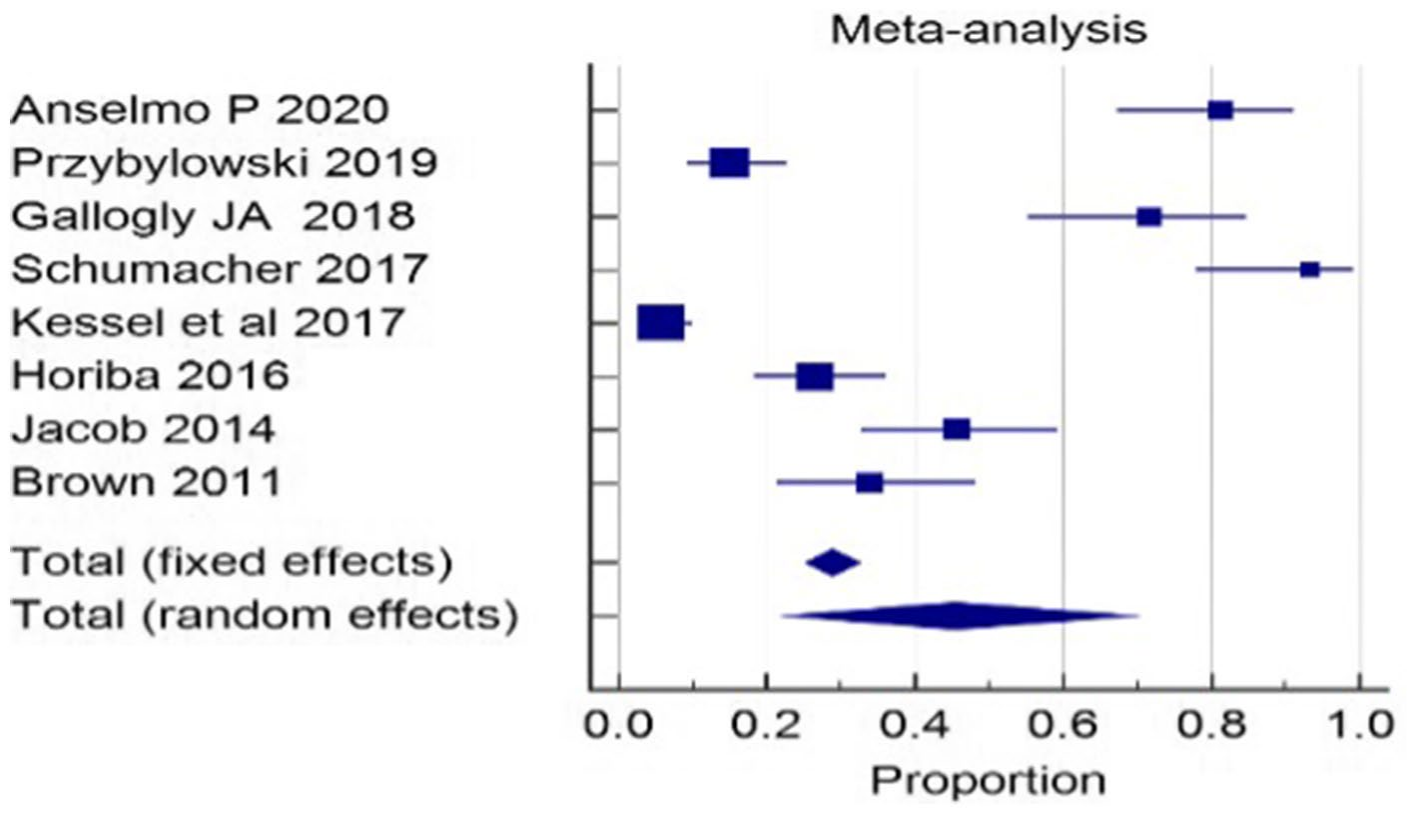
Forest plot for before and after of hearing preservation rate (%).

The diamond is the most prominent element on the plot. It represents the point estimate that sums up all the studies combined. From Figure 1, it can be seen that only three studies lie exactly on the vertical line. This gives an idea about the heterogeneity of the studies. Among the three studies, there was a significant difference in the risk of class A/B, ½ hearing size in comparison to the present and absent (190 participants, RR = 0.44, 95% CI: 0.24–0.82, *p* = 0.009) (Figures 3, 4). From the statistics, it can be seen that I^2^ is 96% which is on the higher side, indicating that the studies are inconsistent for some reason. Ideally, I^2^ should have been <50%. This study is consistent with the findings of Ding et al. (24), who have called for an urgent need for an algorithm to sort the patients with newly diagnosed AN, on the basis of their risks. A funnel plot is also given in Figure 2 to estimate the measure of study precision.

**Figure 5:**
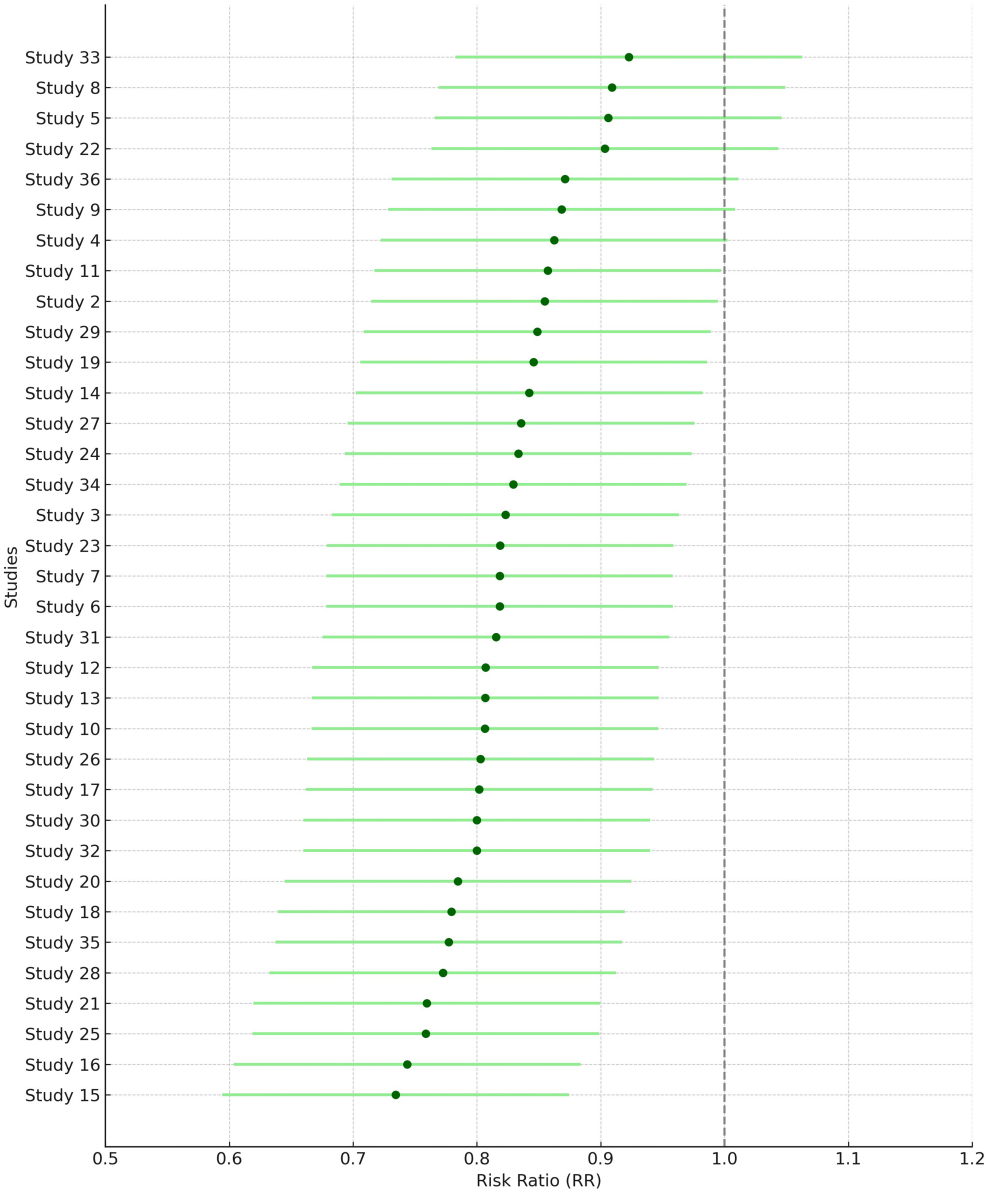
Forest plot of study-level risk ratios for gender-based hearing preservation post-radiotherapy.

**Figure 6:**
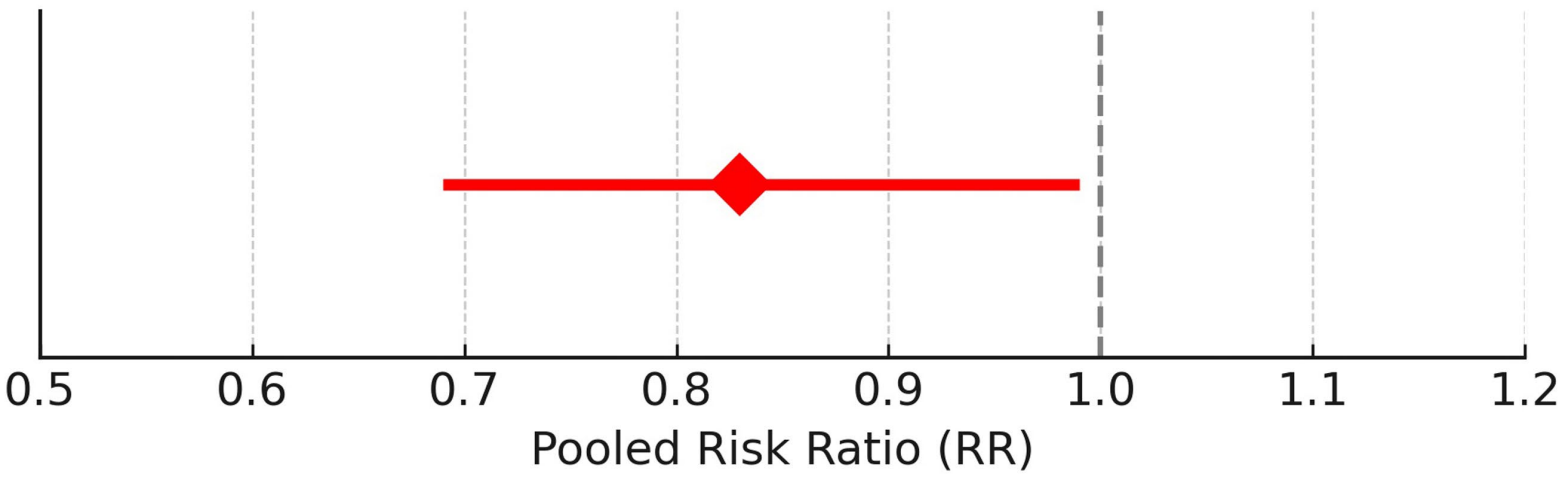
Pooled risk ratio with 95% confidence interval for male vs. female hearing preservation post-radiotherapy.

Gender of the participants was analyzed using a fixed model effect. It was seen that 35 studies showed sex heterogeneity (I^2^ = 70%, *p* = 0.04). The difference in risk levels was significant in comparison to females (3,849 participants, RR = 0.83, 95% CI: 0.69–0.99, *p* = 0.04) (Figures 5, 6). There is hardly any earlier report of a meta-analysis based on gender. Even if they did, the results have not been reported or published due to their insignificance. But here, our studies have reported significant findings based on gender. Figure 2B suggests that males may be at a higher risk of post-radiotherapy hearing loss compared to females; however, this finding should be interpreted with caution. The included studies were observational and did not uniformly adjust for potential confounders such as baseline hearing level, tumor characteristics, or treatment parameters. Therefore, while the pooled estimate reached statistical significance, it does not establish a causal relationship, and further research using adjusted models is warranted to validate this association.

**Figure 7:**
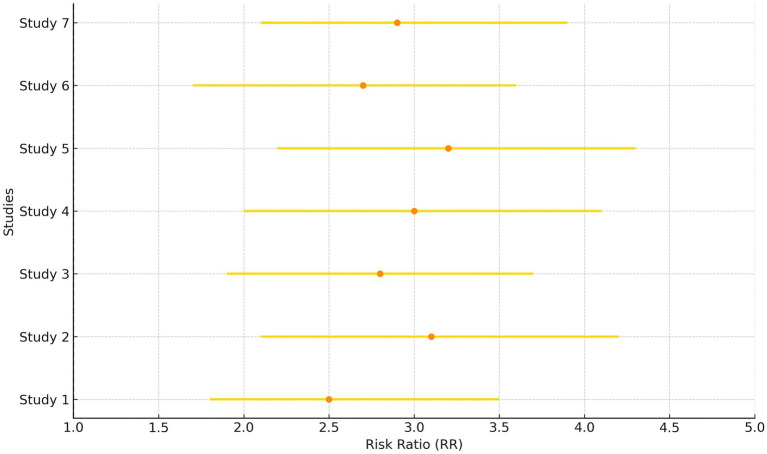
Forest plot of study-level risk ratios for tumor control post-radiotherapy in acoustic neuroma.

**Figure 8:**
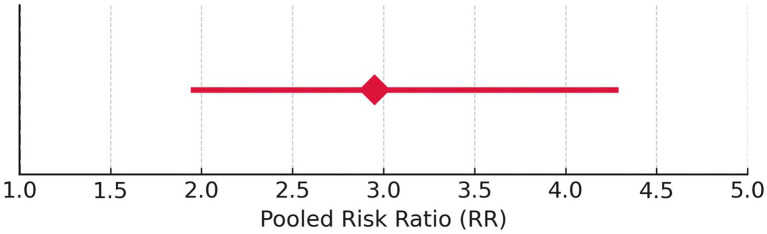
Pooled risk ratio with 95% confidence interval for tumor control post-radiotherapy.

Tumor control rate (%) was analyzed using a fixed model effect. From the forest plot, it can be seen that seven studies exhibit high heterogeneity (I^2^ = 70%, *p* = 0.006). A significant difference was observed in the risk of tumor rate (878 participants, RR = 2.95, 95% CI: 1.94–4.29, *p* = 0.006) (Figures 7, 8). In the majority of the articles, the size of the tumor before and after radiotherapy was not mentioned. Instead, they reported tumor control rates (%). In another similar study with 2,109 patients, in the LC group, the summary effect size was 65% (95% CI: 55.9%; 73.6%), and for the SRS group: 96.9% (95% CI: 94.7%; 98.6%). Overall tumor control showed improvement in the SRS group (*p* < 0.0001) (25). In the majority of the articles considered for the present review, tumor control was successfully achieved. A similar meta-analysis in 2019, on data of 246 patients who opted for SRS or cystic VS with 49.7 to 150 months of follow-up, reported 92% patients with controlled tumor (95% CI: 88–95%). At 5 years, it was 92% (95% CI: 87–95%). By the use of Gamma Knife, a tumor control rate of 93% (95% CI: 88–95%) was achieved. On the basis of data on tumor control rates, they suggested SRS to be a better treatment for cystic AN 21. In another study of 230 patients, the overall tumor control rate, after 46 months of follow-up (range 28–68.8 months), was 93.9% (95% CI: 91.0–96.8%). Here, too, a binary fixed-effects estimate analysis was used (*p* = 0.681, test for heterogeneity) (26). The rate of recurrence of the tumor has been associated with residual tumor volume (27). In another recent meta-analysis, tumor control rate after GKRS was 98% (28), whereas it was 92.7% in another study with 3,233 patients 25. None of the studies had done any analysis on predicting therapy failure.

**Figure 9:**
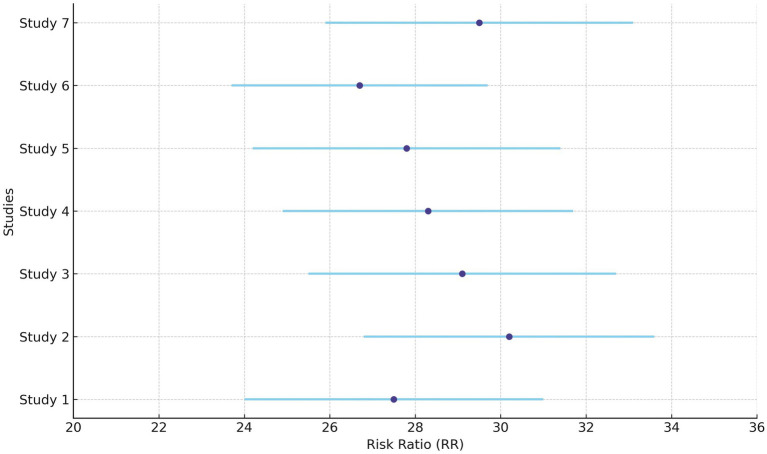
Forest plot of study-level risk ratios for hearing preservation post-radiotherapy in acoustic neuroma.

**Figure 10:**
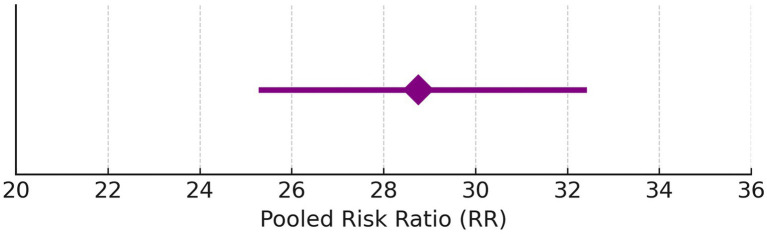
Pooled risk ratio with 95% confidence interval for hearing preservation post-radiotherapy.

Hearing preservation rate (%) was analyzed using a fixed model effect. The heterogeneity observed was high (I^2^ = 97%, *p* < 0.001) in 7 studies. Among the studies, there was a significant difference in the risk of tumor rate (635 participants, RR = 28.76, 95% CI: 25.28–32.42, *p* < 0.001) (Figures 9, 10). The majority of the articles reported hearing preservation rates in the range of 50–90%. Table 3 highlights the variability in hearing preservation rates across studies, with a high degree of heterogeneity (I^2^ = 97.22%), underscoring the need for standardization in outcome reporting and study design. Another recent meta-analysis reported a cochlear nerve preservation rate of 73.4% after surgery, which subsequently decreased to 59.9% at the last follow-up (29). This drop in percentage was reported to be 25% in a study by van de Langenberg et al., and 100% in another study by van de Langenberg et al. (30). These varied percentages of hearing preservation may be due to different pre-therapy rates, which may not be necessarily linked to the size of the tumor. This may be due to the different approaches taken by the treating clinician, for whom hearing preservation may be a primary or a secondary outcome (29). Secondary outcomes were varied, such as imbalance and gait, tinnitus, trigeminal neuralgia, hydrocephalus, ipsilateral facial nerve palsy, thalamic stroke, and transient facial paralysis. However, the percentage of patients who suffered such complications was quite low.

One of the shortcomings of this review is that it does not emphasize analysis based on the time of follow-up since 20–30% patients were observed to have decreasing hearing function at the end of 5 years (31). This might be due to a chronic vascular ischemic mechanism (32). Moreover, the average values of data sets were used for analysis, since none of the included articles revealed the exact data of individual patients. This might have resulted in an unknown variation in the meta-analysis. This analysis also does not take into account hearing loss due to natural or age-related reasons. Some other limitations that are related to individual studies are that a few of them are observational and retrospective in nature. In some studies, there was a lack of standardization in the intervention, reporting of incomplete data, different follow-up periods, different definitions of tumor control rate, different scales to measure hearing loss, and tumor aggravation. Neither any of the individual studies nor the present review looked into predicting factors for hearing loss or failure of treatment.

## Conclusion

The results of this meta-analysis give an insight into the significance and risk factors for gender, tumor control rates, and hearing preservation rates before and after radiotherapy in patients with AN. This information can be useful for patients and clinicians while considering a treatment plan for the benefit of patients. Data from larger studies need to be combined and analyzed especially emphasizing on the follow up time and natural course of hearing loss. The current literature survey and analysis also suggests a long term-controlled study with the use of Hearing Handicap Inventory for Adults to accurately access hearing ability.

## Data Availability

The original contributions presented in the study are included in the article/Supplementary material, further inquiries can be directed to the corresponding author.

## Footnotes

^1^ https://revman.cochrane.org/
