# Supplementary material for: Hearing preservation of post-radiotherapy for acoustic neuroma—a systematic review and meta-analysis
Source: Front Neurol. 2025 Oct 13;16:1647374. doi: 10.3389/fneur.2025.1647374 (PMC12554445; doi:10.3389/fneur.2025.1647374)
Supplement: Supplementary file 1 [file Table_1.DOCX]

# Supplementary Table S1. Newcastle-Ottawa Scale (NOS) Assessment of Included Studies

| **Study** | **Selection (0–4)** | **Comparability (0–2)** | **Outcome (0–3)** | **Total Score (0–9)** | **Quality Rating** | **Mean Age (years)** | **Sample Size** | **Follow-up (months)** | **Hearing Preservation (%)** | **Tumor Control (%)** | **p-value** |
| --- | --- | --- | --- | --- | --- | --- | --- | --- | --- | --- | --- |
| Anselmo et al. (2020) | 4 | 2 | 3 | 9 | High | 61.5 | 48 | 144.0 | 91.0 | 95.0 | 0.002 |
| Franchella et al. (2019) | 3 | 1 | 2 | 6 | Moderate | 47.0 | 19 | 20.5 | 78.0 | 92.0 | 0.034 |
| Tucker et al. (2019) | 4 | 1 | 2 | 7 | High | 63.7 | 52 | 69.0 | 17.3 | 100.0 | 0.008 |
| Przybylowski et al. (2019) | 4 | 2 | 3 | 9 | High | 55.0 | 119 | 49.0 | 59.0 | 88.0 | 0.001 |
| Gallogly et al. (2018) | 3 | 2 | 2 | 7 | High | 53.7 | 40 | 52.3 | 17.5 | 86.4 | 0.045 |
| Deberge et al. (2018) | 3 | 1 | 2 | 6 | Moderate | 59.9 | 142 | 57.0 | 30.9 | 87.0 | 0.023 |
| Ruess et al. (2018) | 4 | 2 | 2 | 8 | High | 58.2 | 335 | 30.0 | 80.0 | 98.0 | 0.003 |
| Schumacher et al. (2017) | 4 | 2 | 3 | 9 | High | 51.0 | 30 | 42.0 | 55.0 | 91.0 | 0.012 |
| Kessel et al. (2017) | 4 | 2 | 2 | 8 | High | 60.0 | 184 | 90.0 | 77.2 | 90.0 | 0.019 |
| Putz et al. (2017) | 3 | 1 | 3 | 7 | High | 62.0 | 107 | 36.0 | 72.0 | 93.0 | 0.027 |

NOS; Newcastle-Ottawa Scale; SD; Standard Deviation; %; Percent; vs.; Versus; p-value; Probability value.
