# Supplementary material for: Hearing preservation of post-radiotherapy for acoustic neuroma—a systematic review and meta-analysis
Source: Front Neurol. 2025 Oct 13;16:1647374. doi: 10.3389/fneur.2025.1647374 (PMC12554445; doi:10.3389/fneur.2025.1647374)
Supplement: Supplementary file 2 [file Table_2.DOCX]

# Supplementary Table S2. Subgroup Analysis of Hearing Preservation Outcomes

| **Subgroup** | **No. of Studies** | **Total Patients** | **Pooled RR** | **95% CI** | **Heterogeneity (I²)** | **Mean Follow-up (months)** | **Hearing Preservation (%)** | **p-value** |
| --- | --- | --- | --- | --- | --- | --- | --- | --- |
| Radiotherapy Type: Gamma Knife | 18 | 2050 | 31.45 | 26.52–37.28 | 72.0 | 54.32 | 68.5 | 0.001 |
| Radiotherapy Type: LINAC | 18 | 1853 | 25.13 | 20.64–30.58 | 68.0 | 56.89 | 61.2 | 0.004 |
| Dose Type: Single-Session | 19 | 2185 | 32.05 | 27.44–37.41 | 74.0 | 52.13 | 70.4 | 0.001 |
| Dose Type: Fractionated | 15 | 1718 | 23.87 | 19.02–29.95 | 65.0 | 59.24 | 59.8 | 0.005 |
| Follow-up Duration: <60 months | 20 | 2220 | 34.11 | 29.45–39.50 | 70.0 | 41.87 | 72.3 | 0.001 |
| Follow-up Duration: ≥60 months | 16 | 1683 | 21.76 | 18.15–26.09 | 66.0 | 75.16 | 55.9 | 0.007 |

RR; Risk Ratio; CI; Confidence Interval; I²; I-squared statistic; LINAC; Linear Accelerator; SD; Standard Deviation; %; Percent; vs.; Versus; p-value; Probability value.
